# Cardiovascular Effects of Nickel: Lippmann et al. Respond

**Published:** 2007-06

**Authors:** Morton Lippmann, Kazuhiko Ito, Polina Maciejczyk, Lung-Chi Chen, Jing-Shiang Hwang

**Affiliations:** New York University School of Medicine, Nelson Institute of Environmental Medicine, Tuxedo, New York, E-mail: lippmann@env.med.nyu.edu; Institute of Statistical Science, Academia Sinica, Taipei, Taiwan

It is readily understandable that Dutton of the International Nickel Company (INCO) would like to separate the nickel emissions from the 381-m INCO smelter stack from the highly statistically significant Ni-associated effects on heart rate and heart rate variability that we observed on 14 of 103 consecutive weekdays of exposure in our laboratory (located within the Sterling Forest State Park) in a mouse model of atherosclerosis (Lippmann et al. 2006). Indeed, it is possible that some closer source of Ni could have been responsible for the effects. However, as we noted in our article, the back trajectories on the 14 days with unusually elevated Ni concentrations did not pass over any known industrial sources or large urban areas, but did pass over or near the distant point source of Ni at the INCO smelter in Sudbury, Ontario, Canada. It is also noteworthy that the 14 back trajectories, illustrated in Figure 2 of our article (Lippmann et al. 2006), approached Sterling Forest from a variety of directions, ranging from the NW to the NNE, making it highly unlikely that that their metals compositions were influenced by a significant source within a 100-mi radius. Furthermore, both the directions of the incoming winds and the combination of unusually high Ni and lower than normal vanadium on those 14 days, as compared with the other 89 days of observation, seemed to preclude the major sources of Ni being the Port of New York, the New York City metropolitan area, or other coastal regions where residual oil is used to generate heat and electrical power. It is well known that residual oil combustion effluents are high in both Ni and V.

We commend Dutton on identifying a mislabeled key in Figure 4 of our article (Lippmann et al. 2006) that occurred during the preparation of the print version. As he noted, the original manuscript published online was correctly labeled. (See the Erratum on p. A294.)

Dutton provided no supporting reference for his comment that “2-year inhalation exposures of rodents to nickel sulfate at levels 600 times higher than those used by Lippmann et al. (2006) were without effect on mortality ….” Based on the 600× concentration difference, we assume he was referring to a National Toxicology Program (NTP) report (NTP 1996). It should be noted that healthy B6C3F_1_ mice were exposed in this study, and that healthy B6 mice had much greater survival times than seven other inbred mice strains when exposed to nickel sulfate by Prows et al. (2003).

Dutton also states that “there is no statistical relationship between NMMAPS [National Mortality and Morbidity Air Pollution Study] CVR [cardiovascular risk] mortality rates and Ni emission rates.” Again, no reference was cited. Even if his statement was supported by a negative study, it relates to emissions and not concentrations. In our article (Lippmann et al. 2006), we showed that the annual average PM_10_ (particulate matter < 10 μm in aerodynamic diameter) NMMAPS mortality coefficients for 60 NMMAPS cities were significantly associated with annual average ambient Ni and V concentrations in those same cities.

Finally, we take exception to Dutton’s comment that implies that we lack sufficient expertise in atmospheric science. The only issue in our article that relates to atmospheric science is our use of HYSPLIT back trajectories, and we fail to see how we misused them.
